# Intestinal Stasis

**Published:** 1915-03

**Authors:** Wm. Arbuthnot Lane

**Affiliations:** Senior Surgeon, Guy's Hospital; Emeritus Surgeon, Hospital for Children, Great Ormond Street, London


*"Scire est nescire, nisi id me
Scire alius sciret."*

MARCH, 1915.

### INTESTINAL STASIS.

BY

SIR WM. ARBUTHNOT LANE, Bart., M.S., F.R.C.S.,[1]

*Senior Surgeon, Guy's Hospital; Emeritus Surgeon, Hospital for Children, Great Ormond Street, London.*

GENTLEMEN,

I appreciate very much the honour you have done me in asking me to address you. I propose to talk about a subject that has engrossed my attention for the last fifteen or sixteen years or more, namely chronic intestinal stasis.

That it is a matter of very great importance is shown by the interest it has aroused, and by the violent opposition to it of all the conservative elements who bulk so largely in our profession. Still, one welcomes it, as criticism stimulates us all to success even more than appreciation. All one asks is that it should not be too unscrupulous in its methods.

[1] An address delivered before the Bristol Medico-Chirurgical Society, March, 1915.

By chronic intestinal stasis I mean such a delay of the contents of the gastro-intestinal tract as results in the absorption into the circulation of a greater amount of poisonous material than the liver, kidneys and skin can deal with. What the substance absorbed is I do not know, and from my point of view it is immaterial, as far as my definition of the condition is concerned. That a perfect knowledge of it is of the greatest importance as regards the treatment of disease is obvious. It must be apparent to the most casual observer that such rough procedures as the operations on the gastro-intestinal tract requisite to stop this infection of the circulation must be replaced or supplemented at no distant date by some simple and scientific method of treatment. At the same time, I feel that we have much to learn before such a happy result ensues.

If the operative procedures which I have devised have done no other good than to stimulate the physician to revise his outlook on medicine, they will have been more than justified.

How beautifully the importance of effective drainage of tissue is illustrated by Carrel's remarkable experiments you all know. I was fortunate to see his experiments on the growth of tissue within the first fortnight of their initiation, and year by year afterwards I saw the same tissue growing uninterruptedly and vigorously under the influence of suitable food and effective drainage.

He shows that any tissue in the body is immortal, provided it is properly fed and properly drained, and that the death of the tissue can only result from defects in food, and especially in drainage. What is true of the tissues is true of the body which they compose, except that the drainage problem in the latter is much more difficult and complex.

You readily realise how important it is to consider our methods of drainage in every particular if you are to prevent the degenerative process which results from imperfect drainage, and the various diseases which develop in our several tissues because of their inability to prevent their inroad by organisms, etc., because their vitality and resisting power have been depreciated. To me that represents the large proportion of the diseases with which we are familiar.

The old drug treatment is gradually disappearing, and most of the operations of the surgeons are for end results, and do not meet the cause of the disease. I do not mean that the removal of the cause may in all cases cure the consequence, but it will do so in the vast majority of cases.

Our present methods of dealing with detail instead of with general principles are to a very large extent futile, and they certainly are most unscientific, and they must gradually be replaced by better ones.

Now we will consider the mechanics of chronic intestinal stasis. The delay starts primarily in the large intestine, which is the cesspool of the gastro-intestinal tract.

In consequence of the accumulation of material in this portion of the intestine it dilates and elongates. This elongation and dilatation are most marked in the first instance in the pelvic colon, and the puddling of this long dilated loop of bowel in the pelvis affords a very great obstacle to the passage of material even under the influence of great abdominal effort ; indeed, the greater the intra-abdominal tension the greater the angulation of the lax bowel.

Now we must clearly understand that the structure of the human body varies in every detail with its mechanical relationship to its surroundings. If the mechanical relationship is altered from the normal, so the structure of the body varies and in direct proportion to the degree in which the mechanical relationship is changing.

This is all most fully illustrated by the remarkable changes which take place during a portion only of the lifetime of the labourer. Many of them I have described in detail in the third edition of my book on *Chronic Intestinal Stasis.* A careful study of these changes will convince the reader of the mechanism of the conditions that arise in the drainage scheme from the constant habitual assumption in civilisation of the erect posture of the trunk except during sleep.

Without a knowledge of the evolutionary changes which develop, the observer too readily (perhaps naturally) assumes that the changes to which I have called attention are inflammatory in origin.

I will describe the mechanical changes as briefly as possible, but before doing so I would point out that any effort that nature makes to meet the peculiar mechanical relationship of the individual to surroundings, while useful for a time, in the end tends to shorten the duration of life. To meet the dilatation, elongation and prolapse of the large intestine, resistances are crystallised along the lines of strain.

These are represented in the first instance by streaks, which form on the peritoneum, which is reflected outwards from the cæcum, ascending colon, hepatic flexure, splenic flexure, descending, iliac, and pelvic colon. These streaks commence at the base of the mesentery, and extend towards the bowel, which they may reach and finally control.

At certain points, especially at the hepatic and splenic flexures, and at the junction of the iliac with the pelvic colon, these bands are more fully developed. They tend to drag the flexures upwards, and to obstruct the lumen of the bowel at these points.

The same anchoring process occurs in an even more marked degree at the brim of the pelvis, where, in the female, these bands frequently attach, surround, and render cystic the left ovary, and also interfere materially with the functioning of the Fallopian tube. This fixation about the junction of the iliac and pelvic colon I have called the " first and last kink," because it is the first to develop in point of time and the lowest in point of place. It is easy to see how the fixation of the left ovary in the anchored bowel is responsible for so much of the pain from which constipated women suffer.

Besides these three points of angulation, the lumen of the large bowel may be constricted elsewhere by a local excessive formation of bands in the mesentery. A volvulus may also develop from the same cause.

This all leads to delay in the passage of material from the cæcum and ascending colon into the transverse colon, and especially to the accumulation and stagnation of fæcal matter in the cæcum. This results in a distension of the cæcum, which drops into the true pelvis, and finally occupies it by displacing all mobile viscera.

The cæcum is suspended externally by a reflection of peritoneum, which extends from its outer and under aspects to the iliac fossa. This is strengthened in the static cæcum by the development of bands, which later form a membrane, first attaching and later suspending the cæcum. The appendix may be secured external to the cæcum by these adhesions, and its lumen being consequently obstructed at one point or another, it may become inflamed, and so-called appendicitis result.

Internally the cæcum is supported by the termination of the ileum and by its mesentery.

This may be strengthened by the formation of streaks, bands, and later membranes in the under surface of the mesentery. These then become attached to the end of the ileum, which they angulate, and whose lumen they obstruct more or less severely. Not uncommonly the appendix may be employed to strengthen this resistance to downward displacement, when it is secured to the under surface of the mesentery.

From its position it presses on the ileum which lies in front of it, and tends to constrict its lumen in the erect posture controlling the ileal effluent. I have called this condition "a controlling appendix."

Again, the anchoring of the appendix in this situation may interfere with the free evacuation of its secretions, and it may become inflamed when the control of the ileal effluent is still further exaggerated by the inflammation of the structure which constricts the ileum mechanically. It is obvious that the material contained in the ileum may be dammed back by the block of the accumulated contents of the cæcum, or it may be produced or accentuated by the "ileal kink," to which American surgeons have attached my name; or by a "controlling appendix," which may be inflamed.

The accumulation of the ileal contents tends to produce a hypertrophy of this bowel, which is most marked in the last two or three inches. The drag exerted by the weight of the loaded small intestines angulates the duodeno-jejunal junction, which from its arrangement is very liable to control.

This causes a dilatation and elongation of the duodenum, whose contents cannot pass into the jejunum, and cannot return through the contracted pylorus to the stomach.

As the first portion of the duodenum is completely surrounded by peritoneum, and unlike the rest of this segment of the bowel is not supported by being attached to adjacent structures, it tends to show greater change in consequence of the excessive distension to which it is exposed. These changes are represented by an engorgement and inflammation, ulceration and perforation of the mucous and other coats of the bowel. The pylorus becomes spasmodically contracted, because of the necessity of the stomach to evacuate its contents, and in order that the duodenal contents shall not return to the stomach. Material, therefore, accumulates in the stomach, which becomes dilated and hypertrophied, and which endeavours to thrust its contents through the obstructing pylorus. In these circumstances the mucous membrane of the lesser curve is exposed to a tearing strain, and it may engorge, inflame and ulcerate. Perforation of the several walls may take place, or the ulcer being very chronic may become cancerous. The mucous membrane about the pylorus, being constantly irritated by the thrust of the stomach contents, may undergo similar changes. These represent what we may call the " mechanical evidences and results of chronic intestinal stasis."

The next results of stasis are those which result from the infection by organisms of the material dammed up for an excessive period of time in the small intestine from which the nutrition of the several tissues of the body is obtained. In other words, we are considering the consequences which ensue on a contamination of the food supply by deleterious organisms.

As a consequence, the biliary and pancreatic ducts become infected by direct extension from the duodenum and indirectly through the blood supply of these organs. As a result the pancreas becomes hard and indurated, it functions imperfectly, and later may become cancerous.

Similarly the liver, overworked and supplied with an impure blood, behaves unsatisfactorily, and becomes liable to many infective processes. Infection of the ducts, the formation of gall-stone, with their consequences terminating not infrequently in cancer, all call for our attention.

The contamination of the several tissues results in the changes I have so frequently described. The thyroid wastes, the adrenal wastes, the breasts become chronically indurated and later cancerous, the skin becomes thin and stained, fat disappears, and the several organs which depend on it largely for support drop and undergo changes in consequence. I refer especially to such organs as the uterus and kidneys. The voluntary and involuntary muscles degenerate. The heart and vessels show very definite changes in consequence of this degenerative process.

The effect on the brain is to produce a varying degree of mental depression, misery, lassitude, headache, inability to work, lunacy and imbecility, sleeplessness, neuritis of various nerves, awaking in the morning feeling no good or rest has resulted from sleep, and a tendency to fall asleep during the daytime are other evidences of the deleterious influence of auto-intoxication upon the brain. Sexual appetite is diminished or destroyed. The secretions of the skin are offensive. There are many other symptoms which are familiar to you all, to which I need not refer now.

In consequence of the depreciation of the vitality, or the lowering of the resisting power of the tissues to the entry of organisms, certain of these effect a foothold, and may after a time produce poisonous products, which aggravate the already existing auto-intoxication. Perhaps the infection that is most obvious and therefore most familiar to us all is the infection of the gums called "pyorrhœa alveolaris." How often does one see a patient's mouth rendered edentulous by the ruthless act of the dentist, acting frequently, I regret to say, on the advice of some medical man, who believes that this infection is a primary cause! How often do patients inform me that they have been assured by eminent physicians that some slight infection of the gums is the cause of all their troubles, and that the removal of the teeth will certainly cure them! All of these unfortunates have been cruelly deceived, and bitterly regret that they were beguiled by the ignorance of their advisers.

Other common infections are rheumatoid arthritis, bronchocele, adenoma of the thyroid, exophthalmic goitre, tubercle, Addison's disease, Still's disease, the various forms of kidney disease, Raynaud's disease, and a host of other conditions which arise in the same way, and which, like the direct conditions of auto-intoxication, are all cured or alleviated by any procedure by which the ileal effluent can be freed from abnormal control, and the food supply freed from infection.

I trust, gentlemen, that I have put this matter before you sufficiently clearly to interest if not to convince you. If I have not succeeded, the limited time at my disposal and the want of the power to impress must be my excuses, not the importance of the subject-matter.

